# Variable clinical phenotype in *TBK1* mutations: case report of a novel mutation causing primary progressive aphasia and review of the literature

**DOI:** 10.1016/j.neurobiolaging.2020.08.014

**Published:** 2021-03

**Authors:** Imogen J. Swift, Martina Bocchetta, Hanya Benotmane, Ione OC. Woollacott, Rachelle Shafei, Jonathan D. Rohrer

**Affiliations:** a UK Dementia Research Institute at University College London, UCL Queen Square Institute of Neurology, University College London, London, UK; b Department of Neurodegenerative Disease, Dementia Research Centre, UCL Queen Square Institute of Neurology, University College London, London, UK

**Keywords:** TBK1, Frontotemporal dementia, Primary progressive aphasia

## Abstract

TANK-binding kinase 1 (*TBK1*) mutations are a recently discovered cause of disorders in the frontotemporal dementia (FTD)–amyotrophic lateral sclerosis (ALS) spectrum. We describe a novel L683∗ mutation, predicted to cause a truncated protein and therefore be pathogenic, in a patient presenting with nonfluent variant primary progressive aphasia at the age of 65 years. Her disease progressed over the following years, leading to her being mute and wheelchair bound seven years into her illness. Brain imaging showed asymmetrical left-sided predominant atrophy affecting the frontal, insular, and temporal cortices as well as the striatum in particular. Review of the literature found 60 different nonsense, frameshift, deletion, or splice site mutations, including the newly described mutation, with data on clinical diagnosis available in 110 people: 58% of the cases presented with an ALS syndrome, 16% with an FTD-ALS overlap, 19% with a cognitive presentation (including behavioral variant FTD and primary progressive aphasia) and 4% with atypical parkinsonism. Age at onset (AAO) data were available in 75 people: mean (standard deviation) AAO was 57.5 (10.3) in those with ALS, which was significantly younger than those with a cognitive presentation (AAO = 65.1 (10.5), *p* = 0.008), or atypical parkinsonism (AAO = 68.3 (8.7), *p* = 0.021), with a trend compared with the FTD-ALS group (AAO = 61.9 (7.0), *p*=0.065); there was no significant difference in AAO between the other groups. In conclusion, clinical syndromes across the whole FTD-ALS-atypical parkinsonism spectrum have been reported in conjunction with mutations in *TBK1*. It is therefore important to include *TBK1* on future gene panels for each of these disorders and to suspect such mutations particularly when there are multiple different phenotypes in the same family.

## Highlights

• Novel *TBK1* mutation, L683∗, is a probable pathogenic mutation in a case of FTD.
• L683∗ lies in the optineurin-binding region, disrupting downstream pathways.
• *TBK1* mutations are found across the entire FTD-ALS-atypical parkinsonism spectrum.
• *TBK1* mutations cause differing ages of onset depending on clinical presentation.

## 1 Introduction

Frontotemporal dementia (FTD) is a heterogeneous disorder commonly presenting with changes in behavior, language, and movement, and characterized by atrophy of the frontal and temporal lobes (Greaves and Rohrer, 2019). FTD can be divided into a behavioral variant (bvFTD) and a language variant, primary progressive aphasia (PPA) (Gorno-Tempini et al., 2011), with PPA being further subtyped into nonfluent (nfvPPA), semantic (svPPA), and logopenic (lvPPA) variants (Gorno-Tempini et al., 2011). However, these canonical behavioral and cognitive syndromes can also overlap with motor disorders including both amyotrophic lateral sclerosis (ALS) and atypical parkinsonism.

FTD is highly heritable, with a genetic component identified in around a third of cases (Rademakers et al., 2012; Rohrer and Warren, 2011). Although most genetic FTD is accounted for by mutations in 3 genes, progranulin (*GRN*), microtubule-associated protein tau (*MAPT*), and chromosome 9 open reading frame 72 (*C9orf72*) (Moore et al., 2020), there are a number of rarer causes including mutations in TANK-binding kinase 1 (*TBK1*), valosin-containing protein, and TAR DNA-binding protein 43 (Greaves and Rohrer, 2019).

**Fig. 1:**
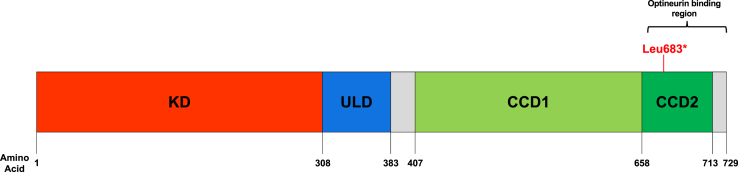
Structure of *TBK1.* The position of the novel mutation described here, Leu683∗, is depicted by the red line and falls in the OPTN binding region (amino acids 661-729). Abbreviations: KD, serine/threonine kinase domain; ULD, ubiquitin-like domain; CCD1, coiled-coil domain 1 (aka scaffold dimerization domain); CCD2, coiled-coil domain 2. (For interpretation of the references to color in this figure legend, the reader is referred to the Web version of this article.)

Mutations in *TBK1* account for around 1%–2% of all FTD, making it probably the fourth most common cause of genetic FTD worldwide (Van Mossevelde et al., 2016). The gene codes for the TBK1 protein, a noncanonical IκB kinase involved in inflammation and autophagy (Ahmad et al., 2016). It is formed of a serine/threonine kinase domain, a ubiquitin-like domain and 2 coiled-coil domains (CCD1 and CCD2) (Fig. 1) and is known to interact with target substrates such as optineurin (OPTN) and interferon regulating factor 3 (Ahmad et al., 2016, Freischmidt et al., 2017, Hu et al., 2018). Pathogenic mutations have been identified in *TBK1* occurring across all 4 domains, with the majority causing haploinsufficiency due to nonsense or frameshift mutations that result in premature termination codons (Freischmidt et al., 2015, Freischmidt et al., 2017, Table 1). Missense variants have also been described although there is some debate about the pathogenicity of these (de Majo et al., 2018).

**Table 1:** Nonsense, deletion, frameshift, and splice site *TBK1* mutations and their associated clinical phenotypes

Mutation			References	Phenotype						
Variant (cDNA) NM_013254.3	Predicted protein change	Mutation alias		bvFTD	nfvPPA	svPPA	CBS	PSP	FTD-ALS	ALS
Kinase domain										
c.4C>T	p.Q2∗	Q2∗	S3, S4, S8, S24, S27	+						+
c.86delA	p.K29Rfs∗15	K29fs	S24		+			+		
c.176dupT	p.L59Ffs∗16	L59fs	S20							+
c.228+1G>A	p.K30_E76del	K30_E76del	S5, S24	+					+	+
c.236_238delCAA	p.T79del	T79del	S4, S5, S8, S24, S26	+					+	+
c.253_255delATT	p.I85del	I85del	S27							+
c.288delT	p.V97Ffs∗2	V97fs	S24							+
c.349C>T	p.R117∗	R117∗	S3, S8, S19, S24	+	+				+	+
c.358+2T>C	p.T77Wfs∗4	T77fs	S6, S17							+
c.379C>T	p.R127∗	R127∗	S24							+
c.467_468delCA	p.T156Rfs∗6	T156fs	S2, S14		+		+		+	+
c.499_501delGAT	p.D167del	D167del	S8, S24, S25							+
c.540+1G>A	p.V120Afs∗3	V120fs	S3							+
c.555T>A	p.Y185∗	Y185∗	S6, S17							+
c.583C>T	p.Q195∗	Q195∗	S6							+
c.621dupT	p.G208Wfs∗15	G208fs	S6							+
c.684dupT	p.R229∗	R229∗	S13							+
c.762_763delTG	p.N254Kfs∗4	N254fs	S5		+				+	+
c.790_791insACAG	p.P264Hfs∗24	P264fs	S6							+
.830delT	p.T278Pfs∗33	T278fs	S7							+
c.834_841delCCCCTGTT	p.P279Cfs∗5	P279fs	S3							+
Ubiquitin-like domain										
c.958delA	p.T320Qfs∗40	T320fs	S6							+
c.992+1G>A	p.G272_T331del	G272_T331del (G>A)	S3, S4, S30	+	+				+	+
c.992+1G>T	p.G272_T331del	G272_T331del (G>T)	S5, S8, S24, S25	+					+	
c.1069C>T	p.R357∗	R357∗	S3, S4, S22							+
Linker region										
c.1192delT	p.S398Pfs∗11	S398fs	S8, S24, S25							+
c.1199delC	p.P400Lfs∗9	P400fs	S15, S29							+
Coiled-coil domain 1										
c.1267_1268delTG	p.C423Lfs∗25	C423fs	S3							+
c.1318C>T	p.R440∗	R440∗	S3, S6, S14						+	+
c.1330C>T	p.R444∗	R444∗	S0, S3, S6, S23						+	+
c.1335G>A	p.W445∗	W445∗	S24		+		+			
c.1340+1G>A	p.A417∗	A417∗	S6, S24	+						+
c.1349_1352delTTAA	p.I450Kfs∗15	I450fs	S6							+
c.1385_1388CAGA	p.T462Kfs∗3	T462fs	S21, S24						+	+
c.1387_1388delGA	p.E463Sfs∗13	E463fs	S3							+
c.1414delA	p.I472Sfs∗8	I472fs	S10							+
c.1432delA	p.T478Lfs∗2	T478fs	S0							+
c.1436_1437delTG	p.V479Efs∗4	V479fs	S6							+
c.1445_1446delAT	p.Y482∗	Y482∗ (1445_1446del)	S13							+
c.1446T>G	p.Y482∗	Y482∗ (1446T>G)	S2, S14			+			+	+
c.1496C>G	p.S499∗	S499∗	S3							+
c.1501_1502insAA	p.I501Kfs∗7	I501fs	S3							+
c.1551_1552insTT	p.S518Lfs∗32	S518fs	S8, S24, S25							+
c.1644-1G>A	p.N548Kfs∗5	N548fs	S18							+
c.1653_1654insA	p.L552Tfs∗23	L552fs	S3							+
c.1760+1G>C	p.R574Sfs∗11	R574fs	S3							+
c.1838T>A	p.L613∗	L613∗	S6							+
c.1852_1854delGAA	p.E618del	E618del	S13							+
c.1869_1875delGCTTCAT	p.M623Ifs∗9	M623fs	S4							+
c.1887_1890delGTTA	p.Q629Hfs∗4	Q629fs	S3, S4							+
c.1928_1930delAAG	p.E643del	E643del	S3, S4, S5, S6, S8, S17, S24, S25, S27, S28	+				+	+	+
c.1959+1_1959+2dupGT		IVS18+1_+2dup	S9			+			+	+
c.1960-2A>G	p.L654Vfs∗18	L654fs	S2, S14		+				+	+
c.1963C>T	p.Q655∗	Q655∗	S2, S14			+			+	+
Coiled-coil domain 2										
c.2040dupT	p.N681∗	N681∗	S13						+	+
c.2048T>G	p.L683∗	L683∗	This paper		+					
c.2063_2064delTT	p.L688Rfs∗14	L688fs	S9	+						+
c.2070delG	p.M690Ifs∗4	M690fs	S1							+
c.2099_2100delTG	p.V700Gfs∗2	V700fs	S16							+
c.2107G>T	p.E703∗	E703∗	S12	+	+		+		+	+
c.2115_2127delCTGAAAATAACCA	p.E706Ffs∗2	E706fs	S0, S11	+						+
c.2138+2T>C	p.M690_R713del	M690_R713del	S6							+

Associated clinical phenotypes shown as + if described for that mutation. In cases of FTD-ALS overlap, the individual FTD syndrome and ALS are also ascribed a + in the phenotype columns. References for each mutation are shown in Supplementary Table 1.

Key: FTD-ALS, frontotemporal dementia–amyotrophic lateral sclerosis; *TBK1,* TANK-binding kinase 1; bvFTD, behavioral variant FTD; nfvPPA, nonfluent variant primary progressive aphasia; svPPA, semantic variant primary progressive aphasia; PSP, progressive supranuclear palsy; CBS, corticobasal syndrome.

The most commonly described phenotype of *TBK1* mutation carriers has been ALS (Table 1). This can present as a pure motor syndrome or with associated cognitive or behavioral deficits that can be mild in some cases or severe enough to meet criteria for FTD-ALS in others (Ahmad et al., 2016; Freischmidt et al., 2017). Pure FTD syndromes have been less commonly reported, but there have been cases described of both bvFTD, and in a smaller number of people, PPA, with both nfvPPA and svPPA phenotypes seen (Caroppo et al., 2015; Jiao et al., 2018; Lamb et al., 2019; Le Ber et al., 2015; van der Zee et al., 2017). In this report, we describe a case of a novel *TBK1* mutation found in a patient presenting with nfvPPA, as well as a review of the literature of the clinical phenotype of patients with *TBK1* mutations.

## 2 Materials and methods

The patient consented to be part of a study at the Dementia Research Centre, UCL Queen Square Institute of Neurology. She underwent a standardized clinical history and examination, neuropsychometric testing, and a 3D T1-weighted magnetic resonance imaging (MRI) on a 3T Siemens Prisma scanner. Using the MRI, we performed a whole-brain parcellation using the geodesic information flow algorithm (Cardoso et al., 2015), which is based on atlas propagation and label fusion. Labels were combined to create volumetric measures of fifteen cortical and 4 subcortical (amygdala, hippocampus, striatum, and thalamus) regions in each hemisphere as well as a brainstem region. All brain volumes were corrected for total intracranial volume, which was calculated using SPM12 (www.fil.ion.ucl.ac.uk/spm). Volumes were expressed as a percentage of 18 age-matched healthy female controls, all scanned on the same MRI scanner: mean (standard deviation) age of controls at scan was 69.2 (6.6) years, versus 70.5 years for the patient. As part of the study, the patient donated a blood sample which underwent whole exome sequencing, and subsequent confirmation of genetic findings using Sanger sequencing.

To review the phenotype of *TBK1* mutation carriers, we performed a PubMed search from the initial description of mutations in *TBK1* in 2015 until May 2020, and identified 31 publications describing nonsense, frameshift, deletion, or splice site mutations that were likely to be pathogenic (Supplementary Table 1). We did not include missense mutations for which there remains some debate over the pathogenicity. To examine whether there were differences in age at onset (AAO) by clinical phenotype within the identified cases, we used a linear regression model in STATA (v.14; TX, USA) with 95% bias-corrected bootstrapped confidence intervals (CIs) with 1000 repetitions.

## 3 Results

### 3.1 Case report

A right-handed woman was initially seen at the age of 69 years with a four-year history of progressive speech difficulties. Her speech had become effortful and nonfluent, with the presence of phonemic errors and agrammatism. She also had binary reversals, often saying “yes” when she meant “no”, and difficulties with understanding long sentences. However, single-word comprehension and semantic knowledge was intact. She had increasing difficulty reading and her writing was affected by agrammatism. There were no memory problems and no difficulties in other cognitive domains apart from mild executive dysfunction in the form of impaired planning and problem solving. There was mild apathy but no other behavioral changes.

She had been otherwise well apart from hypertension, borderline diabetes, and urinary urgency. Her father had died relatively young at the age of 69 years from cancer, but her mother had lived to 85 years, also dying of cancer. Her elder brother had died of cancer at 70 years and she had one younger brother who was alive and well.

Her initial neuropsychometry showed impaired naming, polysyllabic single-word repetition and sentence repetition, as well as executive dysfunction, with other cognitive domains intact (Table 2).

**Table 2:** Neuropsychometric results of the patient at the baseline and follow-up

Test	1st visit	2nd visit
Age at testing	69	70
General intellectual ability		
Mini-Mental State Examination (/30)	NT	25
WAIS-III verbal IQ	NT	54
WAIS-III performance IQ	NT	75
Verbal memory		
Recognition Memory Test for Words	NT	10–25th percentile
Visual Memory		
Topographical Recognition Memory Test	10th percentile	25–50th percentile
Language		
Oldfield Naming Test (/30)	23	5
Sentence repetition (% correct)	20%	0%
Single word repetition		
1-syllable (% correct)	100%	40%
2-syllables (% correct)	82%	30%
3-syllables (% correct)	NT	10%
Test of Reception of Grammar (/40)	NT	27
Concrete Synonyms Test (/25)	NT	15
Praxis		
Limb praxis (% correct)	NT	60%
Orofacial praxis (% correct)	NT	25%
Visuoperceptual and visuospatial skills		
VOSP Shape Detection	>5th percentile	>5th percentile
VOSP Position Discrimination	>5th percentile	>5th percentile
Executive function and processing speed		
Letter fluency (S words in 1 min)	4	1
Category fluency (Animals in 1 min)	5	2
Trail Making Test Part A	10–25th percentile	10–25th percentile
Weigl Sorting Test	<5th percentile	<5th percentile

Key: NT, not tested; VOSP, Visual Object and Space Perception Battery.

<5th percentile is usually considered normal.

**Fig. 2:**
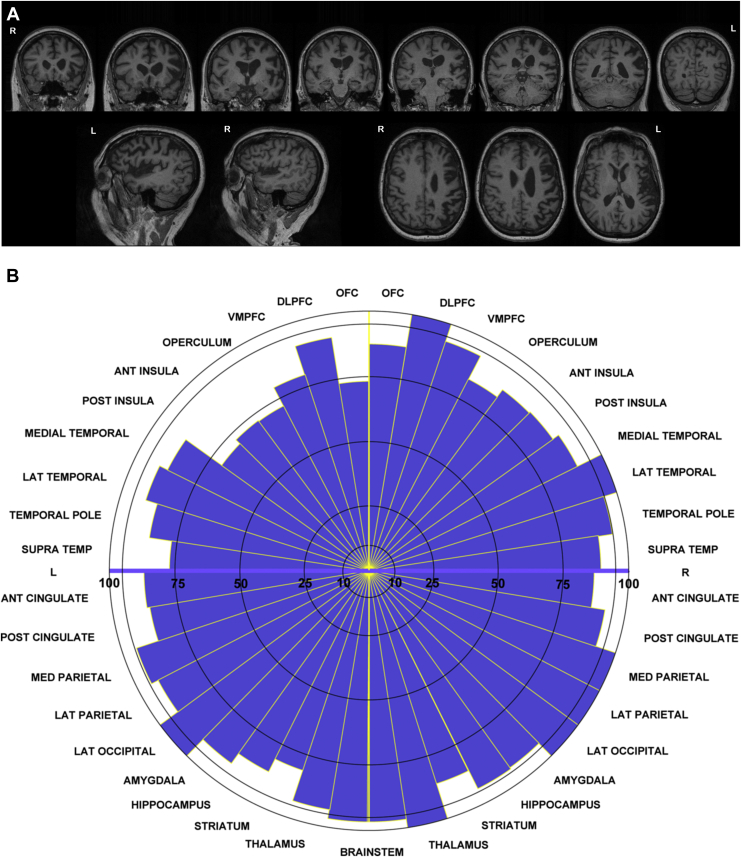
MRI and target diagram showing pattern of atrophy in the patient. (A) T1-weighted images from a 3T MRI scan of the patient at 5.5 years disease duration. Top row: coronal sections; bottom row: sagittal sections (on the left) and axial sections (on the right). Asymmetrical left greater than right hemisphere atrophy is seen with frontal, superior temporal, and parietal involvement. (B) Target diagram showing atrophy seen in different brain regions. Segment length represents the regional volumes of the patient expressed as percentages of the mean volumes of an age-matched healthy control cohort. Abbreviations: OFC, orbitofrontal cortex; DLPFC, dorsolateral prefrontal cortex; VMPFC, ventromedial prefrontal cortex; MED, medial; LAT, lateral; SUPRATEMP, supratemporal cortex; ANT, anterior; POST, posterior.

She subsequently went on to have an MRI brain scan, which showed asymmetrical atrophy affecting the left hemisphere more than the right, with left frontal and insula involvement particularly, but also spreading posteriorly to affect the left temporal and lateral parietal lobes (Fig. 2A). A detailed volumetric analysis confirmed these findings (Fig. 2B) and highlighted involvement also of the subcortical regions, including the striatum.

Overall, the clinical, neuropsychological, and imaging data were consistent with a diagnosis of nfvPPA (Gorno-Tempini et al., 2011).

She underwent genetic testing which found a heterozygous, NM_013254.3: c.2048T>G, p.L683∗, mutation in the *TBK1* gene. This substitution causes a premature stop codon and therefore a truncated protein. No mutations were found in any of the other FTD-associated genes.

### 3.2 Clinical progression

When assessed at the age of 70 years, her speech problems had continued to deteriorate and she had developed difficulties with swallowing. Her walking had slowed down and she was noticed to move her right arm less. On examination at this time, she was found to have a mild right-sided extrapyramidal syndrome with cogwheeling and bradykinesia. There were no features of ALS at this time. By the age of 71 years, she had very little speech output, only able to say “yes” or “no”, with understanding of simple but not complex commands. By 72 years, she was in a wheelchair and mute, and was no longer able to travel to the hospital for review.

### 3.3 Review of clinical phenotype of TBK1 mutation carriers

Including the novel mutation we describe in this article, we identified 60 different nonsense, frameshift, deletion, or splice site mutations (Table 1), with data on clinical diagnosis available in 110 people. 58% of the cases presented with an ALS syndrome. Information on site of onset was available in 19 people, with 63% having limb-onset, 32% bulbar-onset, and 5% respiratory-onset. A further 16% of the total cases presented with an FTD-ALS overlap. The FTD phenotype was mentioned in 12 people: 6 with bvFTD, 3 with nfvPPA (of which 1 case also had a corticobasal syndrome, CBS), and 3 with svPPA. An additional 2% of the total cases had an overlap of an FTD syndrome (1 with nfvPPA and 1 with a PPA syndrome not otherwise specified) with primary lateral sclerosis (Hirsch-Reinshagen et al., 2019).

19% of the total cases had a cognitive presentation. Within this group, 33% had bvFTD and 5% had nfvPPA with 29% having an “FTD” syndrome that was not specified; a further 29% had a dementia syndrome not otherwise specified (often in people in prior generations where a clearer diagnosis was not possible), and 5% a phenotype consistent with Alzheimer's disease (i.e., amnestic onset, Verheijen et al., 2018).

Four percent of cases had an atypical parkinsonian presentation (2 CBS, both overlapping with nfvPPA, and 2 progressive supranuclear palsy [PSP], of which 1 overlapped with nfvPPA). One further case had a progressive cerebellar ataxia (Wilke et al., 2018).

AAO data were available in 75 people. Mean (standard deviation) AAO was 57.5 (10.3) years in those with ALS, which was significantly younger than those with a cognitive presentation where the AAO was 65.1 (10.5) years, mean difference 7.7 (95% CI: 2.0, 13.4), *p* = 0.008. However, this difference was at least in part due to the older AAO in the “dementia-not otherwise specified” group (74.2 (8.4)), with the bvFTD group alone having a similar AAO to the ALS group (57.6 (11.8)). Within the ALS group, there was no difference in AAO between those with limb onset (57.3 (12.2) and those with bulbar onset (56.0 (8.0). There was a trend for the AAO to be older in the FTD-ALS group (61.9 (7.0)) compared with the ALS group, mean difference 4.4 (95% CI: −0.3, 9.1), *p* = 0.065, but there was no difference between the FTD-ALS group and the cognitive group. The AAO in the atypical parkinsonism group was 68.3 (8.7), which was significantly older than the ALS group (mean difference 10.8 (95% CI: 1.7, 19.9), *p* = 0.021) but there was no difference compared with the other groups.

## 4 Discussion

We describe a novel mutation in the *TBK1* gene found in a patient with a diagnosis of nfvPPA, supported by clinical, neuropsychometric, and neuroanatomical findings. No features of ALS were seen during the time the patient was followed up in clinic. This nonsense mutation causes a premature termination codon at position 683, which lies in the CCD2 region of *TBK1* (Freischmidt et al., 2017; Oakes et al., 2017). This region is known to bind to and facilitate the phosphorylation of *OPTN*, an important regulator of autophagy (Freischmidt et al., 2017; Morton et al., 2008; Ryan and Tumbarello, 2018). We propose that this mutation is pathogenic as the truncation of the protein at this position is likely to disrupt the phosphorylation of *OPTN* by *TBK1*, influencing downstream pathways. A previous study has shown disruption to the OPTN/TBK1 interaction in mutations located within the CCD2 region (Li et al., 2016), whereas other studies have shown that protein-truncating mutations in *TBK1* cause reduced TBK1 transcript and/or protein levels, indicative of pathogenicity (Pottier et al., 2015; van der Zee et al., 2017; Yu et al., 2019).

The phenotype of nfvPPA has been reported in a number of other cases previously, although only in 2 prior cases without features of a motor syndrome—other patients have additionally developed ALS, primary lateral sclerosis, CBS, or PSP (Caroppo et al., 2015; Hirsch-Reinshagen et al., 2019; Lamb et al., 2019; Pottier et al., 2015.; van der Zee et al., 2017). A smaller number of patients have been described with an svPPA phenotype (Caroppo et al., 2015), although none have been reported with a lvPPA syndrome. These findings are of clinical importance in terms of potential causes of autosomal dominant PPA: *GRN* mutations are the most common cause of this but mutations in other FTD genes such as *C9orf72* and *MAPT* only very rarely cause PPA (Moore et al., 2020), and so one should include a search for a *TBK1* mutation in those who are *GRN-*negative. Neuroanatomically, as with *GRN* mutations, the pattern of atrophy is often asymmetrical, as described in this case and others (Caroppo et al., 2015; Hirsch-Reinshagen et al., 2019), where there is left more than right, and anterior more than posterior cortical involvement. By contrast, cases presenting with bvFTD and a *TBK1* mutation commonly have asymmetrical right-side predominant atrophy (Koriath et al., 2016; Van Mossevelde et al., 2016).

A review of the phenotypes of patients with *TBK1* mutations reveals that ALS is the predominant clinical syndrome with three-quarters of reported cases having either ALS or ALS combined with an FTD syndrome. Unusually, in the reported cognitive phenotype of FTD-ALS, there were as many cases of PPA (both nfvPPA and svPPA) as there were bvFTD (albeit small numbers in both groups). PPA-ALS, particularly svPPA-ALS is an uncommon phenotype (Greaves and Rohrer, 2019; Tan et al., 2019), and this finding suggests a role for TBK1 in the development of this overlap syndrome.

Atypical parkinsonism is a rare phenotype with only 4% of cases having either a CBS (2%) or PSP syndrome (2%). CBS is usually sporadic but rarely can be caused by mutations in *MAPT* or *GRN*, whereas PSP is almost always sporadic with *MAPT* mutations a very rare cause (Moore et al., 2020). Nonetheless, in patients with an autosomal dominant history of atypical parkinsonism, *TBK1* should be part of any atypical parkinsonism gene panel along with *MAPT* and *GRN*, and mutations in *TBK1* suspected if other family members have ALS (which would not be seen in *MAPT* or *GRN* mutations).

This is the first study to our knowledge to bring together data on AAO across different clinical phenotypes. Although numbers still remain small, data suggest earlier onset in pure ALS syndromes compared with joint ALS-cognitive syndromes as well as pure cognitive or parkinsonian disorders. Future research will be helpful to understand whether there are differences in age at death and disease duration as well, as is seen in *C9orf72* expansions where ALS causes a shorter disease process and therefore earlier age at death (Moore et al., 2020).

In summary, we describe a novel *TBK1* mutation found in a patient presenting with nfvPPA. We propose that this mutation is pathogenic, leading to a truncated protein and affecting the ability of TBK1 to bind to OPTN. Clinical syndromes across the FTD-ALS-atypical parkinsonism spectrum have been reported, and so mutations in *TBK1* should always be kept in mind with these presentations, particularly in families where there are multiple different phenotypes.

## Disclosure statement

The authors declare that they have no conflict of interest.

None of the authors' institutions have contracts relating to this research through which it or any other organization may stand to gain financially now or in the future.

None of the authors or their institutions hold any other agreements that could be seen as involving a financial interest in this work.

All appropriate approval and procedures were used concerning human subjects and animals.

## CRediT authorship contribution statement

**Imogen J. Swift:** Conceptualization, Methodology, Writing - original draft. **Martina Bocchetta:** Writing - review & editing, Resources, Data curation. **Hanya Benotmane:** Data curation, Writing - review & editing. **Ione OC. Woollacott:** Investigation, Writing - review & editing. **Rachelle Shafei:** Investigation, Writing - review & editing. **Jonathan D. Rohrer:** Conceptualization, Methodology, Writing - review & editing, Visualization, Supervision, Funding acquisition.
