# Supplementary material for: Variable clinical phenotype in TBK1 mutations: case report of a novel mutation causing primary progressive aphasia and review of the literature
Source: Neurobiol Aging. 2021 Mar;99:100.e9–100.e15. doi: 10.1016/j.neurobiolaging.2020.08.014 (PMC7907669; doi:10.1016/j.neurobiolaging.2020.08.014)
Supplement: Supplementary Table 1 [file mmc1.docx]

**Supplementary Table 1 – List of references identified in Pubmed search describing nonsense, deletion, frameshift and splice site mutations in TBK1.**

| S0 | Black HA, Leighton DJ, Cleary EM, Rose E, Stephenson L, Colville S, Ross D, Warner J, Porteous M, Gorrie GH, Swingler R, Goldstein D, Harms MB, Connick P, Pal S, Aitman TJ, Chandran S. [Genetic epidemiology of motor neuron disease-associated variants in the Scottish population.](https://pubmed.ncbi.nlm.nih.gov/28089114/?from_term=chandran+mnd&from_sort=date&from_size=100&from_pos=21) Neurobiol Aging. 2017 Mar;51:178.e11-178.e20. |
| --- | --- |
| S1 | Borghero G, Pugliatti M, Marrosu F, Marrosu MG, Murru MR, Floris G, Cannas A, Occhineri P, Cau TB, Loi D, Ticca A, Traccis S, Manera U, Canosa A, Moglia C, Calvo A, Barberis M, Brunetti M, Gibbs JR, Renton AE, Errichiello E, Zoledziewska M, Mulas A, Qian Y, Din J, Pliner HA, Traynor BJ, Chiò A; ITALSGEN and SARDINALS Consortia. [TBK1 is associated with ALS and ALS-FTD in Sardinian patients.](https://pubmed.ncbi.nlm.nih.gov/27156075/?from_term=%238+OR+%236&from_sort=date&from_size=100&from_exact_term=%28%28TBK1+FTD%29+OR+%28TBK1+ALS%29%29&from_pos=75) Neurobiol Aging. 2016 Jul;43:180.e1-5. |
| S2 | Caroppo P, Camuzat A, De Septenville A, Couratier P, Lacomblez L, Auriacombe S, Flabeau O, Jornéa L, Blanc F, Sellal F, Cretin B, Meininger V, Fleury MC, Couarch P, Dubois B, Brice A, Le Ber I. Semantic and nonfluent aphasic variants, secondarily associated with amyotrophic lateral sclerosis, are predominant frontotemporal lobar degeneration phenotypes in TBK1 carriers. Alzheimers Dement (Amst). 2015 Oct 30;1(4):481-6. |
| S3 | Cirulli ET, Lasseigne BN, Petrovski S, Sapp PC, Dion PA, Leblond CS, Couthouis J, Lu YF, Wang Q, Krueger BJ, Ren Z, Keebler J, Han Y, Levy SE, Boone BE, Wimbish JR, Waite LL, Jones AL, Carulli JP, Day-Williams AG, Staropoli JF, Xin WW, Chesi A, Raphael AR, McKenna-Yasek D, Cady J, Vianney de Jong JM, Kenna KP, Smith BN, Topp S, Miller J, Gkazi A; FALS Sequencing Consortium, Al-Chalabi A, van den Berg LH, Veldink J, Silani V, Ticozzi N, Shaw CE, Baloh RH, Appel S, Simpson E, Lagier-Tourenne C, Pulst SM, Gibson S, Trojanowski JQ, Elman L, McCluskey L, Grossman M, Shneider NA, Chung WK, Ravits JM, Glass JD, Sims KB, Van Deerlin VM, Maniatis T, Hayes SD, Ordureau A, Swarup S, Landers J, Baas F, Allen AS, Bedlack RS, Harper JW, Gitler AD, Rouleau GA, Brown R, Harms MB, Cooper GM, Harris T, Myers RM, Goldstein DB. [Exome sequencing in amyotrophic lateral sclerosis identifies risk genes and pathways.](https://pubmed.ncbi.nlm.nih.gov/25700176/?from_term=%238+OR+%236&from_sort=date&from_size=100&from_exact_term=%28%28TBK1+FTD%29+OR+%28TBK1+ALS%29%29&from_pos=88) Science. 2015 Mar 27;347(6229):1436-41. |
| S4 | de Majo M, Topp SD, Smith BN, Nishimura AL, Chen HJ, Gkazi AS, Miller J, Wong CH, Vance C, Baas F, Ten Asbroek ALMA, Kenna KP, Ticozzi N, Redondo AG, Esteban-Pérez J, Tiloca C, Verde F, Duga S, Morrison KE, Shaw PJ, Kirby J, Turner MR, Talbot K, Hardiman O, Glass JD, de Belleroche J, Gellera C, Ratti A, Al-Chalabi A, Brown RH, Silani V, Landers JE, Shaw CE. [ALS-associated missense and nonsense TBK1 mutations can both cause loss of kinase function.](https://pubmed.ncbi.nlm.nih.gov/30033073/?from_term=%238+OR+%236&from_sort=date&from_size=100&from_exact_term=%28TBK1+FTD%29+OR+%28TBK1+ALS%29&from_pos=31) Neurobiol Aging. 2018 Nov;71:266.e1-266.e10. |
| S5 | Dols-Icardo O, García-Redondo A, Rojas-García R, Borrego-Hernández D, Illán-Gala I, Muñoz-Blanco JL, Rábano A, Cervera-Carles L, Juárez-Rufián A, Spataro N, De Luna N, Galán L, Cortes-Vicente E, Fortea J, Blesa R, Grau-Rivera O, Lleó A, Esteban-Pérez J, Gelpi E, Clarimón J. [Analysis of known amyotrophic lateral sclerosis and frontotemporal dementia genes reveals a substantial genetic burden in patients manifesting both diseases not carrying the C9orf72 expansion mutation.](https://pubmed.ncbi.nlm.nih.gov/28889094/?from_term=%238+OR+%236&from_sort=date&from_size=100&from_exact_term=%28%28TBK1+FTD%29+OR+%28TBK1+ALS%29%29&from_pos=48) J Neurol Neurosurg Psychiatry. 2018 Feb;89(2):162-168. |
| S6 | Freischmidt A, Wieland T, Richter B, Ruf W, Schaeffer V, Müller K, Marroquin N, Nordin F, Hübers A, Weydt P, Pinto S, Press R, Millecamps S, Molko N, Bernard E, Desnuelle C, Soriani MH, Dorst J, Graf E, Nordström U, Feiler MS, Putz S, Boeckers TM, Meyer T, Winkler AS, Winkelman J, de Carvalho M, Thal DR, Otto M, Brännström T, Volk AE, Kursula P, Danzer KM, Lichtner P, Dikic I, Meitinger T, Ludolph AC, Strom TM, Andersen PM, Weishaupt JH. [Haploinsufficiency of TBK1 causes familial ALS and fronto-temporal dementia.](https://pubmed.ncbi.nlm.nih.gov/25803835/?from_term=%238+OR+%236&from_sort=date&from_size=100&from_exact_term=%28%28TBK1+FTD%29+OR+%28TBK1+ALS%29%29&from_pos=87) Nat Neurosci. 2015 May;18(5):631-6. |
| S7 | Garton FC, Benyamin B, Zhao Q, Liu Z, Gratten J, Henders AK, Zhang ZH, Edson J, Furlong S, Morgan S, Heggie S, Thorpe K, Pfluger C, Mather KA, Sachdev PS, McRae AF, Robinson MR, Shah S, Visscher PM, Mangelsdorf M, Henderson RD, Wray NR, McCombe PA. [Whole exome sequencing and DNA methylation analysis in a clinical amyotrophic lateral sclerosis cohort.](https://pubmed.ncbi.nlm.nih.gov/28717666/?from_term=garton+als&from_sort=date&from_size=100&from_pos=6) Mol Genet Genomic Med. 2017 Jun 12;5(4):418-428. |
| S8 | Gijselinck I, Van Mossevelde S, van der Zee J, Sieben A, Philtjens S, Heeman B, Engelborghs S, Vandenbulcke M, De Baets G, Bäumer V, Cuijt I, Van den Broeck M, Peeters K, Mattheijssens M, Rousseau F, Vandenberghe R, De Jonghe P, Cras P, De Deyn PP, Martin JJ, Cruts M, Van Broeckhoven C; BELNEU Consortium. [Loss of TBK1 is a frequent cause of frontotemporal dementia in a Belgian cohort.](https://pubmed.ncbi.nlm.nih.gov/26581300/?from_term=%238+OR+%236&from_sort=date&from_size=100&from_exact_term=%28%28TBK1+FTD%29+OR+%28TBK1+ALS%29%29&from_pos=80) Neurology. 2015 Dec 15;85(24):2116-25. |
| S9 | Jiao B, Sun Q, Yuan Z, Wang J, Zhou L, Yan X, Tang B, Shen L. Rare TBK1 variants in patients with frontotemporal dementia and amyotrophic lateral sclerosis in a Chinese cohort. Transl Neurodegener. 2018 Dec 4;7:31. |
| S10 | Kim YE, Oh KW, Noh MY, Nahm M, Park J, Lim SM, Jang JH, Cho EH, Ki CS, Lee S, Kim SH. [Genetic and functional analysis of TBK1 variants in Korean patients with sporadic amyotrophic lateral sclerosis.](https://pubmed.ncbi.nlm.nih.gov/27939697/?from_term=%238+OR+%236&from_sort=date&from_size=100&from_exact_term=%28%28TBK1+FTD%29+OR+%28TBK1+ALS%29%29&from_pos=61) Neurobiol Aging. 2017 Feb;50:170.e1-170.e6. |
| S11 | Koriath CA, Bocchetta M, Brotherhood E, Woollacott IO, Norsworthy P, Simón-Sánchez J, Blauwendraat C, Dick KM, Gordon E, Harding SR, Fox NC, Crutch S, Warren JD, Revesz T, Lashley T, Mead S, Rohrer JD. [The clinical, neuroanatomical, and neuropathologic phenotype of TBK1-associated frontotemporal dementia: A longitudinal case report.](https://pubmed.ncbi.nlm.nih.gov/28229125/?from_term=%238+OR+%236&from_sort=date&from_size=100&from_exact_term=%28%28TBK1+FTD%29+OR+%28TBK1+ALS%29%29&from_pos=57) Alzheimers Dement (Amst). 2016 Nov 3;6:75-81. |
| S12 | Lamb R, Rohrer JD, Real R, Lubbe SJ, Waite AJ, Blake DJ, Walters RJ, Lashley T, Revesz T, Holton JL, Morris HR. [A novel TBK1 mutation in a family with diverse frontotemporal dementia spectrum disorders.](https://pubmed.ncbi.nlm.nih.gov/31160356/?from_term=%238+OR+%236&from_sort=date&from_size=100&from_exact_term=%28TBK1+FTD%29+OR+%28TBK1+ALS%29&from_pos=16) Cold Spring Harb Mol Case Stud. 2019 Jun 3;5(3):a003913. |
| S13 | Lattante S, Doronzio PN, Marangi G, Conte A, Bisogni G, Bernardo D, Russo T, Lamberti D, Patrizi S, Apollo FP, Lunetta C, Scarlino S, Pozzi L, Zollino M, Riva N, Sabatelli M. [Coexistence of variants in TBK1 and in other ALS-related genes elucidates an oligogenic model of pathogenesis in sporadic ALS.](https://pubmed.ncbi.nlm.nih.gov/31000212/?from_term=%238+OR+%236&from_sort=date&from_size=100&from_exact_term=%28TBK1+FTD%29+OR+%28TBK1+ALS%29&from_pos=20) Neurobiol Aging. 2019 Dec;84:239.e9-239.e14. |
| S14 | Le Ber I, De Septenville A, Millecamps S, Camuzat A, Caroppo P, Couratier P, Blanc F, Lacomblez L, Sellal F, Fleury MC, Meininger V, Cazeneuve C, Clot F, Flabeau O, LeGuern E, Brice A; French Clinical and Genetic Research Network on FTLD/FTLD-ALS. [TBK1 mutation frequencies in French frontotemporal dementia and amyotrophic lateral sclerosis cohorts.](https://pubmed.ncbi.nlm.nih.gov/26476236/?from_term=%238+OR+%236&from_sort=date&from_size=100&from_exact_term=%28%28TBK1+FTD%29+OR+%28TBK1+ALS%29%29&from_pos=82) Neurobiol Aging. 2015 Nov;36(11):3116.e5-3116.e8. |
| S15 | McCann EP, Williams KL, Fifita JA, Tarr IS, O’Connor J, Rowe DB, Nicholson GA, Blair IP. The genotype-phenotype landscape of familial amyotrophic lateral sclerosis in Australia. Clin Genet. 2017 Sep;92(3):259-266. |
| S16 | McCombe PA, Ngo ST, Guo CC, Fazlollahi A, Bollmann S, Wang L, Hu X, Barth M, Salvado O, Davis M, Ceslis A, Robinson G, Henderson RD, Steyn FJ. [Patient with ALS with a novel TBK1 mutation, widespread brain involvement, behaviour changes and metabolic dysfunction.](https://pubmed.ncbi.nlm.nih.gov/30196251/?from_term=%238+OR+%236&from_sort=date&from_size=100&from_exact_term=%28TBK1+FTD%29+OR+%28TBK1+ALS%29&from_pos=28) J Neurol Neurosurg Psychiatry. 2019 Aug;90(8):952-954. |
| S17 | Müller K, Brenner D, Weydt P, Meyer T, Grehl T, Petri S, Grosskreutz J, Schuster J, Volk AE, Borck G, Kubisch C, Klopstock T, Zeller D, Jablonka S, Sendtner M, Klebe S, Knehr A, Günther K, Weis J, Claeys KG, Schrank B, Sperfeld AD, Hübers A, Otto M, Dorst J, Meitinger T, Strom TM, Andersen PM, Ludolph AC, Weishaupt JH; German ALS network MND-NET. [Comprehensive analysis of the mutation spectrum in 301 German ALS families.](https://pubmed.ncbi.nlm.nih.gov/29650794/?from_term=%238+OR+%236&from_sort=date&from_size=100&from_exact_term=%28%28TBK1+FTD%29+OR+%28TBK1+ALS%29%29&from_pos=35) J Neurol Neurosurg Psychiatry. 2018 Aug;89(8):817-827. |
| S18 | Naruse H, Ishiura H, Mitsui J, Date H, Takahashi Y, Matsukawa T, Tanaka M, Ishii A, Tamaoka A, Hokkoku K, Sonoo M, Segawa M, Ugawa Y, Doi K, Yoshimura J, Morishita S, Goto J, Tsuji S. [Molecular epidemiological study of familial amyotrophic lateral sclerosis in Japanese population by whole-exome sequencing and identification of novel HNRNPA1 mutation.](https://pubmed.ncbi.nlm.nih.gov/29033165/?from_term=naruse+tbk1&from_sort=date&from_size=100&from_pos=2) Neurobiol Aging. 2018 Jan;61:255.e9-255.e16. |
| S19 | Pottier C, Bieniek KF, Finch N, van de Vorst M, Baker M, Perkersen R, Brown P, Ravenscroft T, van Blitterswijk M, Nicholson AM, DeTure M, Knopman DS, Josephs KA, Parisi JE, Petersen RC, Boylan KB, Boeve BF, Graff-Radford NR, Veltman JA, Gilissen C, Murray ME, Dickson DW, Rademakers R. [Whole-genome sequencing reveals important role for TBK1 and OPTN mutations in frontotemporal lobar degeneration without motor neuron disease.](https://pubmed.ncbi.nlm.nih.gov/25943890/?from_term=%238+OR+%236&from_sort=date&from_size=100&from_exact_term=%28%28TBK1+FTD%29+OR+%28TBK1+ALS%29%29&from_pos=84) Acta Neuropathol. 2015 Jul;130(1):77-92. |
| S20 | Pozzi L, Valenza F, Mosca L, Dal Mas A, Domi T, Romano A, Tarlarini C, Falzone YM, Tremolizzo L, Sorarù G, Cerri F, Ferraro PM, Basaia S, Agosta F, Fazio R, Comola M, Comi G, Ferrari M, Quattrini A, Lunetta C, Penco S, Bonanomi D, Carrera P, Riva N. [TBK1 mutations in Italian patients with amyotrophic lateral sclerosis: genetic and functional characterisation.](https://pubmed.ncbi.nlm.nih.gov/28822984/?from_term=%238+OR+%236&from_sort=date&from_size=100&from_exact_term=%28%28TBK1+FTD%29+OR+%28TBK1+ALS%29%29&from_pos=51) J Neurol Neurosurg Psychiatry. 2017 Oct;88(10):869-875. |
| S21 | Schönecker S, Brendel M, van der Zee J, van Broeckhoven C, Rominger A, Danek A, Levin J. [[A Pair of Siblings with Frontotemporal Dementia and Amyotrophic Lateral Sclerosis and a Novel Thr462Lysfs Mutation in the TBK1 Gene].](https://pubmed.ncbi.nlm.nih.gov/27570907/?from_term=%238+OR+%236&from_sort=date&from_size=100&from_exact_term=%28%28TBK1+FTD%29+OR+%28TBK1+ALS%29%29&from_pos=66) Fortschr Neurol Psychiatr. 2016 Aug;84(8):494-8. |
| S22 | Tohnai G, Nakamura R, Sone J, Nakatochi M, Yokoi D, Katsuno M, Watanabe H, Watanabe H, Ito M, Li Y, Izumi Y, Morita M, Taniguchi A, Kano O, Oda M, Kuwabara S, Abe K, Aiba I, Okamoto K, Mizoguchi K, Hasegawa K, Aoki M, Hattori N, Onodera O, Naruse H, Mitsui J, Takahashi Y, Goto J, Ishiura H, Morishita S, Yoshimura J, Doi K, Tsuji S, Nakashima K, Kaji R, Atsuta N, Sobue G; Japanese Consortium for Amyotrophic Lateral Sclerosis Research (JaCALS). [Frequency and characteristics of the TBK1 gene variants in Japanese patients with sporadic amyotrophic lateral sclerosis.](https://pubmed.ncbi.nlm.nih.gov/29398122/?from_term=%238+OR+%236&from_sort=date&from_size=100&from_exact_term=%28%28TBK1+FTD%29+OR+%28TBK1+ALS%29%29&from_pos=38) Neurobiol Aging. 2018 Apr;64:158.e15-158.e19. |
| S23 | Tsai PC, Liu YC, Lin KP, Liu YT, Liao YC, Hsiao CT, Soong BW, Yip PK, Lee YC. [Mutational analysis of TBK1 in Taiwanese patients with amyotrophic lateral sclerosis.](https://pubmed.ncbi.nlm.nih.gov/26804609/?from_term=%238+OR+%236&from_sort=date&from_size=100&from_exact_term=%28%28TBK1+FTD%29+OR+%28TBK1+ALS%29%29&from_pos=79) Neurobiol Aging. 2016 Apr;40:191.e11-191.e16. |
| S24 | van der Zee J, Gijselinck I, Van Mossevelde S, Perrone F, Dillen L, Heeman B, Bäumer V, Engelborghs S, De Bleecker J, Baets J, Gelpi E, Rojas-García R, Clarimón J, Lleó A, Diehl-Schmid J, Alexopoulos P, Perneczky R, Synofzik M, Just J, Schöls L, Graff C, Thonberg H, Borroni B, Padovani A, Jordanova A, Sarafov S, Tournev I, de Mendonça A, Miltenberger-Miltényi G, Simões do Couto F, Ramirez A, Jessen F, Heneka MT, Gómez-Tortosa E, Danek A, Cras P, Vandenberghe R, De Jonghe P, De Deyn PP, Sleegers K, Cruts M, Van Broeckhoven C, Goeman J, Nuytten D, Smets K, Robberecht W, Damme PV, Bleecker J, Santens P, Dermaut B, Versijpt J, Michotte A, Ivanoiu A, Deryck O, Bergmans B, Delbeck J, Bruyland M, Willems C, Salmon E, Pastor P, Ortega-Cubero S, Benussi L, Ghidoni R, Binetti G, Hernández I, Boada M, Ruiz A, Sorbi S, Nacmias B, Bagnoli S, Sorbi S, Sanchez-Valle R, Llado A, Santana I, Rosário Almeida M, Frisoni GB, Maetzler W, Matej R, Fraidakis MJ, Kovacs GG, Fabrizi GM, Testi S. [TBK1 Mutation Spectrum in an Extended European Patient Cohort with Frontotemporal Dementia and Amyotrophic Lateral Sclerosis.](https://pubmed.ncbi.nlm.nih.gov/28008748/?from_term=%238+OR+%236&from_sort=date&from_size=100&from_exact_term=%28%28TBK1+FTD%29+OR+%28TBK1+ALS%29%29&from_pos=60) Hum Mutat. 2017 Mar;38(3):297-309. |
| S25 | Van Mossevelde S, van der Zee J, Gijselinck I, Engelborghs S, Sieben A, Van Langenhove T, De Bleecker J, Baets J, Vandenbulcke M, Van Laere K, Ceyssens S, Van den Broeck M, Peeters K, Mattheijssens M, Cras P, Vandenberghe R, De Jonghe P, Martin JJ, De Deyn PP, Cruts M, Van Broeckhoven C; Belgian Neurology consortium. [Clinical features of TBK1 carriers compared with C9orf72, GRN and non-mutation carriers in a Belgian cohort.](https://pubmed.ncbi.nlm.nih.gov/26674655/?from_term=van+mossevelde+TBK1&from_sort=date&from_size=100&from_pos=4) Brain. 2016 Feb;139(Pt 2):452-67. |
| S26 | Verheijen J, van der Zee J, Gijselinck I, Van den Bossche T, Dillen L, Heeman B, Gómez-Tortosa E, Lladó A, Sanchez-Valle R, Graff C, Pastor P, Pastor MA, Benussi L, Ghidoni R, Binetti G, Clarimon J, de Mendonça A, Gelpi E, Tsolaki M, Diehl-Schmid J, Nacmias B, Almeida MR, Borroni B, Matej R, Ruiz A, Engelborghs S, Vandenberghe R, De Deyn PP, Cruts M, Van Broeckhoven C, Sleegers K; BELNEU Consortium; EU EOD Consortium. [Common and rare TBK1 variants in early-onset Alzheimer disease in a European cohort.](https://pubmed.ncbi.nlm.nih.gov/29146049/?from_term=%238+OR+%236&from_sort=date&from_size=100&from_exact_term=%28%28TBK1+FTD%29+OR+%28TBK1+ALS%29%29&from_pos=45) Neurobiol Aging. 2018 Feb;62:245.e1-245.e7. **One case with a phenotype of Alzheimer’s disease (T79del).** |
| S27 | Weinreich M, Shepheard SR, Verber N, Wyles M, Heath PR, Highley JR, Kirby J, Shaw PJ. [Neuropathological characterization of a novel TANK binding kinase (TBK1) gene loss of function mutation associated with amyotrophic lateral sclerosis.](https://pubmed.ncbi.nlm.nih.gov/31498468/?from_term=%238+OR+%236&from_sort=date&from_size=100&from_exact_term=%28TBK1+FTD%29+OR+%28TBK1+ALS%29&from_pos=12) Neuropathol Appl Neurobiol. 2020 Apr;46(3):279-291. |
| S28 | Wilke C, Baets J, De Bleecker JL, Deconinck T, Biskup S, Hayer SN, Züchner S, Schüle R, De Jonghe P, Synofzik M. Beyond ALS and FTD: the phenotypic spectrum of TBK1 mutations includes PSP-like and cerebellar phenotypes. Neurobiol Aging. 2018 Feb;62:244.e9-244.e13. **One case with a phenotype of cerebellar ataxia (E643del).** |
| S29 | Williams KL, McCann EP, Fifita JA, Zhang K, Duncan EL, Leo PJ, Marshall M, Rowe DB, Nicholson GA, Blair IP. Novel TBK1 truncating mutation in a familial amyotrophic lateral sclerosis patient of Chinese origin. Neurobiol Aging. 2015 Dec;36(12):3334.e1-3334.e5. |
| S30 | Hirsch-Reinshagen V, Alfaify OA, Hsiung GR, Pottier C, Baker M, Perkerson RB 3rd, Rademakers R, Briemberg H, Foti DJ, Mackenzie IR. [Clinicopathologic correlations in a family with a *TBK1* mutation presenting as primary progressive aphasia and primary lateral sclerosis.](https://pubmed.ncbi.nlm.nih.gov/31244341/?from_term=rademakers+foti&from_sort=date&from_pos=1)Amyotroph Lateral Scler Frontotemporal Degener. 2019 Nov;20(7-8):568-575. |
